# Time Signature Detection: A Survey

**DOI:** 10.3390/s21196494

**Published:** 2021-09-29

**Authors:** Jeremiah Abimbola, Daniel Kostrzewa, Pawel Kasprowski

**Affiliations:** Department of Applied Informatics, Silesian University of Technology, 44-100 Gliwice, Poland; jeremiah.oluwagbemi.abimbola@polsl.pl (J.A.); pawel.kasprowski@polsl.pl (P.K.)

**Keywords:** time signature, meter, metre, measure signature, music information retrieval, signal processing, deep learning

## Abstract

This paper presents a thorough review of methods used in various research articles published in the field of time signature estimation and detection from 2003 to the present. The purpose of this review is to investigate the effectiveness of these methods and how they perform on different types of input signals (audio and MIDI). The results of the research have been divided into two categories: classical and deep learning techniques, and are summarized in order to make suggestions for future study. More than 110 publications from top journals and conferences written in English were reviewed, and each of the research selected was fully examined to demonstrate the feasibility of the approach used, the dataset, and accuracy obtained. Results of the studies analyzed show that, in general, the process of time signature estimation is a difficult one. However, the success of this research area could be an added advantage in a broader area of music genre classification using deep learning techniques. Suggestions for improved estimates and future research projects are also discussed.

## 1. Introduction

The majority of popular music scores are composed in a particular style known as lead sheet format. It summarizes a song by representing the notes of the main theme, the chord series, and other cues such as style, tempo, and time signature. Symbols are used in standard staff music notation to denote note duration (onset and offset times). Onset notes refers to the beginning of a notation or another sound and all musical notes have an onset, but do not always contain the first transient [1]. Offset is about the duration of the part from the beginning of the piece. It is the sum of the previous duration only when there is no rest and there are no place where two notes play together [1,2]. In addition, the staff contains details about the tempo, the beginning, the end of the bars, and the time signature. The time signature (sometimes referred to as a meter signature or metre signature) is a symbol for western music which specifies the number of beats (pulses) in each measure (bar) [3]. It is defined as a ratio of two integer numbers, where the numerator indicates the number of beats in a bar and the denominator specifies the note relation [4]. There are simple and compound time signatures that are relatively easy to estimate from the lead sheet or audio files. Examples include $\frac{2}{2}$, $\frac{3}{4}$, $\frac{4}{4}$, or $\frac{6}{8}$ which means 2 minim beats, 3 crotchet beats, 4 crochets beats, and 6 quaver beats in a bar, respectively. The compound signatures are a multiples of the simple time signatures in terms of the number of beats [5]. Examples include $\frac{6}{8}$, $\frac{9}{8}$, $\frac{12}{8}$. There are also irregular time signatures that are much more difficult to estimate [6]. Examples include $\frac{5}{8}$, $\frac{7}{8}$, and $\frac{11}{8}$. Time signature estimation and detection cannot be possible without understanding the concept of upbeat, downbeat, and anacrusis. Upbeats and downbeats represent the simplest manner of associating downward motions with melodic movements to metrically stable points [7]. “Down” beats are times of stronger metric stability in the field of meter [8]. Anacrusis was defined by Lerdahl and Jackendoff [9,10] in two ways: “from the start of the group to the most powerful beat inside a group” and “from an upbeat to its related downbeat”. With anacrusis in mind, estimating the time signature becomes tougher when the first note of the piece is not the strongest note. Additionally, the idea of strong beats must be considered in this process because strong beat spectrum peaks will occur during repetition moments in highly organized or repeated music. This shows both pace and relative intensity of certain beats, so that different types of rhythms may be distinguished at the same timing [11,12,13].

The music industry is fast-growing and with songs being produced every day, curators like Apple Music, Spotify, Audio Mack, etc. need a genre classification system to accurately curate playlist for their users. This involves grouping musical data together based on defined similarities such as rhythm—time signature and tempo—or harmonic content. In this domain, many attempts have been made to classify various genres of songs with notable successes [14,15,16,17]. However, with extracted features, such as time signature, the overall accuracy could get even better but this area is comparatively unexplored by reason of the estimation being difficult.

Estimating time signature is a challenging task because all the beat times after the downbeat (strong beat) before the next downbeat do not always correspond to the total number of beats in a bar, especially for audio music. The main reason for this is because the tempo of any music track affects the time signature significantly. Beat times here refers to the time in seconds for a beat to sound relative to the entire duration of the track. For example, a track of 80 bpm with beat times as 1.02, 2.03, 3.03, 4.02 could estimate as $\frac{4}{4}$; 1.02 being the downbeat time, whereas the same track played with 120 bpm could have beat times as 1.02, 1.33, 1.82, 2.13 which absolutely cannot be estimated as a $\frac{4}{4}$ if it is assumed that a second beat time corresponds to a beat. Therefore, a couple of factors need to be put into consideration to accurately estimate the time signature, namely upbeat, downbeat, anacrusis, onset note, and tempo. However challenging, the automatic detection of time signature could help to reduce computational time for other temporal processes such as beat tracking, tempo estimation, and deep learning techniques. Moreover, it can be a preprocessing step to other tasks, such as gathering knowledge about music, automatic tagging of songs, improving genre classification, and recommendation systems.

Understanding time signature and its function in feature extraction, estimation, and music genre classification would open the door to new possibilities for the music information retrieval domain [18,19]. Feature extraction for audio signal analysis is one of the most important steps for further studies related to time signature detection [20]. YAAFE [21], an audio feature extraction software, was developed in 2010 which has features including, but not limited to, speech/music discrimination, music genre, or mood recognition, and as a result, there has been improvement over the years. Garima Sharma et al. [22] also highlights the trends of various methods that have been used to extract audio signal features.

A significant amount of study has been conducted on the automated retrieval of meta data from musical audio signals. Pitch detection [23,24], onset detection [25,26], key signature estimate [27], and tempo extraction [28,29] are some of the meta data obtained by various algorithms. The aim is two-fold: to equip computers with the capabilities of a human music listener in order to interpret a piece of music and derive explanations of specific musical properties. This enables a variety of applications, including automated transcription, playlist generation, and Music Information Retrieval (MIR) systems as is always discussed at every International Symposium on Music Information Retrieval (ISMIR) [30].

The algorithms that have been employed so far can be divided into two major approaches: the classical and the deep learning approach. The classical or manual approach involves using methods in the digital signal processing domain as evident in [31] by Meinard et al. in a study that showed how a piece of music can be analyzed by signal processing, by using comb filters [32] proposed by Klapuri just to mention a few. In an attempt to estimate time signature, one must have a sound knowledge about concepts such as frequency, tones, notes, duration, timbre, audio spectrum, beats, tempo, and timing. The deep learning approach, on the other hand, makes use of deep learning models. There are ideas that are common to both methods such as the use of Fourier transforms and analysis and the conversion of audio signals to log spectrograms—a more scientifically usable form. It is important to note that the majority of these approaches were implemented using MATLAB [33,34,35] and C++ for collaborations, testing and method validation up until around 2015 and beyond that Python became the go-to. This interest was sparked by several reasons, such as ease of understanding the language and the availability of high-quality machine study libraries, like scikit-learn [36] and librosa [37], just to name a few.

This paper summarizes and reviews numerous research in this field, taking into account similar works, datasets, and a possible road map. To the best of our knowledge, no paper has ever conducted a survey on time signature detection or estimation. As a result, it is important that this survey be conducted in order to identify potential paths for creative ideas. Additionally, in this study, in the course of exploration, a deeper knowledge of frequencies in the time domain is obtained which may be useful in other domain areas like medicine and psychology, which have referred to beats as the pulse of the heart.

The study is organized as follows. Section 2 discusses the music input signals and their impact on the methodologies. In Section 3, datasets utilized in this domain are highlighted in depth. In Section 4, state-of-the-art classical approaches are described, while in Section 5, deep learning approaches are examined in depth. Section 6 concludes the paper with a review of the results achieved and the proposed future course for this domain.

## 2. Musical Input Signals

Time signature estimation can be carried out by using two types of input data: music audio samples or Musical Instrument Digital Interface (MIDI) signals. The music audio samples basically refer to compressed sample files like mp3, uncompressed files like wav, or any other audio format usable with a range of roughly 20 to 20,000 Hz, which corresponds to the lower and upper limits of human hearing. For example, the audio signal on a compact disc is limited to a maximum frequency of 20 kHz, sampled at 44.1 kHz and encoded, with 16 bits per sample [38] and nearly perfect audio signals are obtained with 64 kb/s [38]. Sampling in music refers to the use of a part (or sample) of a sound file of another recording. Samples can be layered, equalized, sped up or slowed down, re-pitched, looped, or otherwise manipulated and can include elements such as rhythm, harmony, voice, vibrations, or whole bars of music [31].

On the other hand, the MIDI is a standard digital interface for communication with a musical instrument and other associated audio devices for performing, editing, and recording music [39]. A MIDI music piece’s sound quality is determined by the synthesizer (sound card), and has other restrictions, such as the inability to save voice, which takes up far less space, making it much easier to store, share, adjust, and manipulate as well as being universally accepted and allowing for greater comparison between music works played on various instruments [40]. This is why some researchers prefer this format. A summary of their differences is shown in Table 1. As a result, this section has been divided into two subsections in order to properly understand how these input signals have impacted previous studies.

### 2.1. Audio Samples as Data

**Figure 1:**
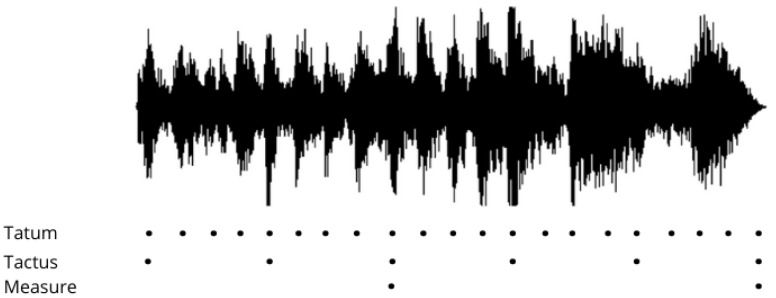
An audio signal with three metrical levels illustrated: tatum, tactus, and measure levels.

An audio signal can be analyzed at three levels in a time scale as discovered by Klapuri et al. [41]: at the temporally atomic tatum pulse level, the tactus pulse level that corresponds to a piece’s tempo, and the harmonic measure level as shown in Figure 1. Christian Uhle et al. [42] as the pioneers of this research area in 2003, were very much interested in estimation and detection of three basic rhythm features: tempo, micro-time, and time signature in which musical pieces can be partly characterized by. The estimation of these three features was combined and individually separated by the integer ratios between them. The process involved the decomposition of four-second audio signal samples into frequency bands, a high-pass filter was applied—as the human ear cannot perceive sounds below 20 Hz [43], half-wave rectified amplitude envelopes were used to track onsets notes, and the filtered signal envelopes of each band were removed.

The inter-onset intervals (IOIs) are then determined from the note onset times, and the tatum duration is measured using an IOI histogram. Using an auto-correlation system, periodicities in the temporal progression of the amplitude envelopes are observed in the subsequent processing. The auto-correlation function peaks refer to the time lags at which the signal is most close to itself. The envelopes of two segments are accumulated in advance, allowing for the measurement of a bar duration of up to four seconds. This estimate is inspired by the assumptions that self-similarity exists at the tatum, beat, and bar levels [44]. The algorithm’s [42] output was evaluated using 117 samples 8-second-long each of percussive music. Music from different backgrounds and cultures, such as West African, Brazilian, and Japanese folkloristic music, and solo drum-set performance, were included in the test results. The presented research technique calculated tempo, micro time, and time signature from percussive music. A total of 84.6 percent of the tempo values, 83.8 percent of the micro duration, and 73.5 percent of the time signatures were accurately measured from 117 quotations of eight seconds length. However, this approach does not explicitly gives the estimation in terms of the numerator and denominator of the time signature which is our main focus.

### 2.2. MIDI Signals as Data

Nowadays, the MIDI signals are not really used anymore for this task because technology has provided better options, however, they were famously used back then because they are easier to work with owing to the precise signal patterns. For instance, the detection of onset notes can be obtained more precisely [11,45] because of the patterns that exist among the notes and a lot of researchers have exploited this advantage. Although it would be outstanding if a DAW like Logic Pro X can automatically determine the time signature by dragging a MIDI file into it, today, this is not common practice as MIDI data can adapt to any tempo and time signature specified. Grohganz et al. in [46] showed that the musical beat and tempo information is often defined in the MIDI files at a preset value that is not associated with the actual music content, so they introduced the method for determining musical beat grids in the provided MIDI file. They also showed, as a major addition, how the global time signature estimate may be utilized to fix local mistakes in the Pulse Grid estimate. Unlike the digital audio signal, when the notes are not perfectly on the grid, they could be quantized first before any process of time estimation is done.

The assumption that the MIDI track is repetitive almost throughout the song was also used by Roig et al. in [47], and similar to the ASM, the Rhythm Self Similarity Matrix (RSSM) was employed for this study. In order to construct the RSSM using the tactus as a measuring unit, the rhythmic elements will be divided into the number of tactus corresponding to their duration. As a result, the inter onset interval (IOI) of each note is separated into tactus intervals.

## 3. Datasets

In every classification, estimation, or detection project, the dataset selection is critical. Sometimes, there are a range of potentially viable datasets available, each with their own set of advantages and drawbacks, and the decision to choose one dataset over another may have a huge impact on the project’s outcome [48]. The journey of obtaining robust and well-balanced datasets has seen a shift from a very simple set to attempts at providing larger and more diverse datasets as shown in Table 2.

The RWC dataset [49] was one of the first set of datasets that was put together solely for academic purposes. Shared libraries that made important contributions to scientific advancements were popular in other fields of scholarly study. It includes six original collections: the Popular Music Database (100 songs), the Royalty-Free Music Database (15 songs), the Classical Music Database (50 pieces), the Jazz Music Database (50 pieces), the Music Genre Database (100 pieces), and the Musical Instrument Sound Database (50 instruments). The data files of this dataset consist of audio signals, corresponding regular MIDI archives, and text files with lyrics all totaling 365 musical pieces performed and recorded. It also takes account of individual sounds at half-tone intervals with a variety of playing techniques, dynamics, instrument makers, and musicians. This collection served as a baseline to which researchers tested and analyzed different structures and methods. Unfortunately, this dataset is very small and unbalanced.

Six years later, Ju-Chiang Wang et al. created another dataset, CAL500 [50], for music auto-tagging as an improvement of the RWC datasets with about 502 songs but the audio files are not provided in the dataset. The tag labels are annotated in the segment level instead of the track level. Unfortunately, 502 songs is inadequate to get better and accurate results for auto-tagging.

The evolution of datasets in the music domain or music information retrieval space cannot be discussed without mentioning the GTZAN dataset [51] collected by G. Tzanetakis and P. Cook. It is by far the most popular dataset out there containing 1000 song excerpts of 30 s, sampling rate 22,050 Hz at 16 bit collected from various sources including personal CDs, radio, microphone recordings, and so on. Its songs are distributed evenly into 10 different genres: Blues, Classical, Country, Disco, Hip Hop, Jazz, Metal, Pop, Reggae, and Rock. Since its publication in 2002, the GTZAN has been widely used in music genre classification analysis [58,59,60,61,62]. It was selected mostly because it was well-organized and widely quoted in previous studies. This precedent lends authority while also providing a frame of reference for results. However, there are a few disadvantages to using this dataset. Its relatively small size is the most limiting factor.

Mandel and Ellis created USPOP [52], centered only on popular artists with over 8752 audio songs without the raw file provided. Obviously, this is not a good dataset as its skewing can be questioned. Skewed datasets usually have a very high impact on the solutions they are used for as highlighted in these studies [63,64,65].

Chris Hartes, in 2010, created the Beatles datatset [66] which contains 180 songs and was well annotated by the musicologist Alan W. Pollack. Each music recording contains on average 10 sections from 5 unique section-types. It was one of the datasets used to generate the Million Song Dataset.

Another notable dataset is SWAT10K [67]. This dataset was obtained from the Echo Nest API in conjunction with Pandora, having 10,870 audio songs that are weakly labeled using a tag vocabulary of 475 acoustic tags and 153 genre tags with the files also not provided. For developers and media firms, the Echo Nest is a music intelligence and data platform located in Somerville, MA bought by Spotify in 2014. The Echo Nest originated as an MIT Media Lab spin-off to investigate the auditory and textual content of recorded music. Its designer’s intentions for the APIs are for music recognition, recommendation, playlist construction, audio fingerprinting, and analysis for consumers and developers [68]. Pandora is a subscription-based music streaming service headquartered in Oakland, California. It focuses on suggestions based on the “Music Genome Project”, a method of categorizing individual songs based on musical characteristics. Like the SWAT10K, MagnaTagATune [54] which has 25,863 audio files provided as csv was also created based on the Echo Nest API. Another dataset for popular music is the MusicCLEF [56] with 200,000 audio songs provided for research purpose.

The Free Music Archive (FMA) by Defferrard et al. [55] contains over 100,000 tracks, each with its own genre label. There are many variations of the dataset available, ranging from the *small* version (8000 30-S samples) to the *full* version (all 106,574 songs in their entirety). The size of this dataset makes it suitable for labeling, and the fact that the audio files are available for download ensures that features can be derived directly from the audio.

The Million Song Dataset (MSD) [57] is a set of audio features and metadata for a million contemporary songs (as the name implies) that is publicly accessible. Release year, artist, terms of the artist, related artists, danceability, energy, length, beats, tempo, loudness, and time signature are among the metadata and derived features included in the dataset although audio files with proper tag annotations (top-50 tags) are only available for about 240,000 previews of 30 s [69].

A very recent dataset, Augmented Maps (A-MAPS) [70] was created in 2018 with no precise number of MIDI files specified. However, it is the most common dataset used for automatic transcription of music. Adrien Ycart et al. updated the previous version of the original MIDI files, containing onset, offsets, and additional annotations. The annotations include duration of notes in fraction relative to a $\frac{1}{4}$th note (a crotchet), tempo curve, time signature, key signature (annotated as a relative major key), separate left and right-hand staff, and text annotations from the score (tempo indications, coda). However, due to MIDI format constraints, they do not contain all of the details required for staff-notation music transcription. It is difficult to say how this dataset was obtained because the original dataset MAPS is not readily available at the time of writing this paper.

Among all these datasets, having seen their advantages and drawbacks, the two that seem very useful to this review in terms of time signature extraction are the FMA and the Million Song Dataset which are both extracted from the Echo Nest API. However, the metadata from the MSD have been pre-processed which makes it difficult to know how it was carried out, although there is a confidence level for the data we are most interested in (time signature).

## 4. Classical Methods

**Figure 2:**
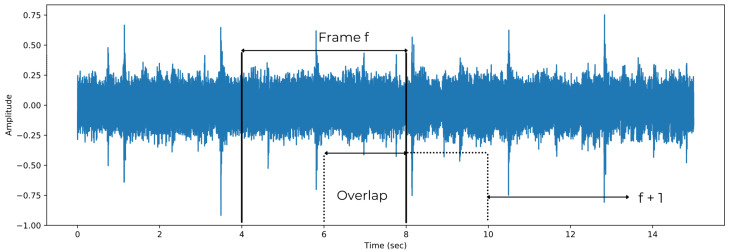
One of the most common methods of audio preprocessing—splitting the whole signal into frames with overlapping.

The methods discussed in this section consists of digital signal processing of audio samples tasks such as window framing in Figure 2, filtering and Fourier analysis [71]. Audio tracks are usually divided into perceivable audio chunks known as frames where 1 sample at 44.1 KHz is 0.0227 ms. This time is far shorter than what the human ear can meaningfully resolve—10 ms. Therefore in order to avoid spectral leakage, a windowing function is applied which eliminates samples at both ends of the frame hence the importance of the frame overlap to have a continuous signal again. Some of these processes will be explained in detail and a brief summary can be found in Table 3.

Aggelos Pikrakis et al. in [73] presented an extraction method for time signature which was referred to as meter. This method was also based on the assumption that the music meter was constant throughout the audio signals. Their assumption was valid given the type of music they used for the estimation—300 raw audio samples of Greek traditional dance music whose tempo ranges from 40 bpm to 330 bpm. It is important to note that there is a huge relationship between the speed of any music track (in bpm) and the time signature, as pointed out by Lee in [80]. By considering a similar approach as the ASM, a self-similarity matrix was used for this experiment which showed that periodicities corresponding to music meter and beat are revealed at the diagonals of the matrix of the audio spectrogram. Consequently, by examining these periodicities, it is possible to estimate both meter and beat simultaneously. In the first step, each raw audio recording was divided into non-overlapping long-term segments with a length of 10 s each. The meter and tempo of the music were removed segment by segment. A short-term moving window, in particular, produces a series of function vectors for each long-term fragment. The approximate values for the short-term window duration and overlap duration between successive windows are 100 ms and 97 ms, implying a 3 ms moving window phase. The overall result accounted for the successful extraction of the rhythmic features while most mistaken results were produced for meters such as 2/4 with 4/4 or 5/4; 7/8 with 3/4 or 4/4.

Since the ASM method proved to be effective, Gainza in [76] combined it with a Beat Similarity Matrix to estimate the meter of audio recordings. To begin, a spectrogram (a pictorial representation of the power of a signal or “loudness” of a signal over time at different frequencies of a specific waveform [81]) of the audio signal was generated using windowed frames with a length of L=1024 samples and a hop size of H=512 samples, which is half the frame length. Then, individual audio similarity matrices were calculated by comparing the spectrogram frames of the piece of music every two beats. Following that, a beat similarity matrix was constructed by combining similarity measures obtained from the individual audio similarity matrices. Finally, by processing the diagonals of the beat similarity matrix, the presence of identical patterns of beats was studied. The equation to obtain the matrix diagonals is defined as

(1) $$X\left(m,k\right)=abs\left[\sum_{n=0}^{L-1}x\left(n+mH\right)w{\left(n\right)}^{*}e^{-j(2/\tau /N)k.n}\right]$$

w(n) is a windowing function which in this case is the Hanning window that selects a *L* length block from the input signal x(n), and *m*, *N*, *H*, and *k* are the frame index, fast Fourier transform (FFT) length, hop size, and bin number respectively; k∈{1:N/2}. The choice of the window type function was based on previous studies [82,83]. The findings obtained demonstrate the robustness of the presented approach, with 361 songs from a database of quadruple meters, a database of truple meters, and another of complex meters yielding a 95% accuracy.

Furthermore, Gouyon and Herrera in [72] proposed a method to determine the meter of music audio signals by seeking recurrences in the beat segment. Several approaches were considered with the aim of testing the hypothesis that acoustic evidence for downbeats can be calculated on signal low-level characteristics, with an emphasis on their temporal recurrences. One approach is to determine which of the low-level audio features corresponding to specific meters were relevant for meter detection. This approach is limited because it was simplified to two-groupings only (duple and triple group meters) while not considering the cases for irregular meters. With a frame size of 20 ms and a hop size of 10 ms, features such as energy, spectral flatness, and energy in the upper half of the first bark band were extracted from each signal frame. Beat segmentation was also carried out as a different approach based on these features already extracted. For the study, a database of 70 sounds (44,100 Hz, 16 bit, mono) was used. Each extract is 20 s long. Bars for beginnings and endings were set at random, and music from Hip-hop, Pop, Opera, Classical, Jazz, Flamenco, Latin, Hard-rock, and other genres were included.

As a more advanced technique to this problem, they also considered the classification methods to assign the value for the meter: from a non-parametric model (Kernel Density estimation) to a parametric one (Discriminant Analysis), including rule induction, neural networks, 1-Nearest Neighbor (1-NN), or Support Vector Machines (SVMs). For this, on a frame-by-frame basis, the following features were computed: energy, zero-crossing rate, spectral centroid, spectral kurtosis, spectral skewness, two measures of spectral flatness (one is the ratio geometric mean/arithmetic mean and the other is the ratio harmonic mean/arithmetic mean), 13 Mel-Frequency Cepstrum Coefficients (MFCCs), and energy in 26 non-overlapping spectral bands. The evaluation showed that, when 27 features were used, error rates for all cases were found to be less than 17.2% (the best technique, Naive Bayes, yielded just 5.8%, whereas a rule induction technique yielded 17.2%).

**Figure 3:**
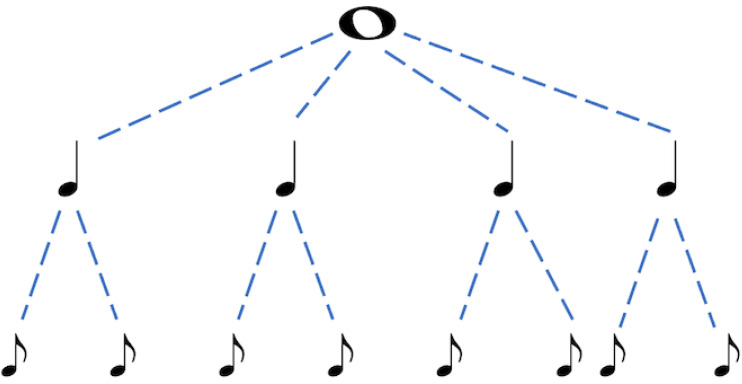
The hierarchical tree structure of notes—the metrical structure of a $\frac{4}{4}$ bar (1 whole note = 4 quarter notes = 8 eighth notes).

Meter detection was also studied from the aspect of breaking down the metrical structure of a single bar by Andrew and Mark in [84] using some excerpts from Bach which eventually gave a 80.50% F-measure. They started by using the hierarchical tree structure of notes as seen in Figure 3. This gave insight for evaluation on each of the three levels (sub-beat, beat, and bar) of the guessed metrical tree. If it matched exactly a level of the metrical tree, it was counted as a true positive and otherwise, a clash was counted as a false positive. In another study [85], they pushed this model furthermore to accurately detect the meter. The suggested model was based on two musicological theories: a reasonably steady rate of the tatum without great discontinuities and notes that are relatively similar to those tatums. Each state in the model represents a single bar, with a list of tatums from that bar and a metrical hierarchy defining which tatums are beats and sub-beats. The tatum list and the downbeat of the next bar are obtained. The tatums are listed in ascending chronological order. The metrical hierarchy of a state has a certain number of tatums per sub-beat, sub-beats per beat, and beats per bar, as well as an anacrusis duration, which is determined by the number of tatums that fall before the first downbeat of a given piece. The first downbeat position probability was also considered by De Haas et al. [86] with a model—Inner Metric Analysis (IMA). The number of tatum per sub-beat was restricted to 4. Although, in principle, this could be any number. The set of possible sub-beat per beat and beat per bar pairs (i.e., time signatures) are taken all of those found in our training set ($\frac{2}{X}$, $\frac{3}{X}$, $\frac{4}{X}$, $\frac{6}{X}$, $\frac{9}{X}$, and $\frac{12}{X}$), where *X* could be any value ranging from 1 to 12.

Gulati et al. then took on this very difficult challenge to estimate the meter of irregular time signature using the case study of Indian classical music in their study with meters of 7/8 [77]. The incoming audio stream is transformed to a mono channel after being downsampled to 16 kHz. The data is divided into 32 ms frames with a 5 ms hop size and a frame rate of 200 Hz. Each frame is subjected to a Hamming window, and a 512-point FFT is calculated. With 12 overlapping triangle filters that are equally spaced on the Mel-frequency scale, the frequency bins are reduced to 12 non-linear frequency bands. The time history of the amplitudes of each of these 12 bands is represented by a band envelope with a sampling frequency of 200 Hz (frame rate). The band envelope is then transformed to log scale (dB) and low pass filtered using a half-wave raised cosine filter. The meter vector $\vec{m}$ is obtained when narrow comb filter banks are set up around integer multiples of tatum duration retrieved from the differential signal. The number of comb filters implemented per filter bank is equal to twice the integer multiple of the tatum duration plus one to account for the tatum duration’s round-off factor. For each filter bank, the filter with the maximum output energy (i.e., with a certain delay value) is chosen, and the total energy of this filter over all Mel bands is calculated. The salience value for each feasible meter is calculated in Equations (2)–(4) i.e., for double, triple, and septuple. A simple rule-based technique is used to calculate the final meter value from $\vec{m}$.

(2) $$S_{2}=\left[\vec{m}\left(4\right)+\vec{m}\left(8\right)+\vec{m}\left(16\right)\right]\cdot \frac{1}{3}$$

(3) $$S_{3}=\left[\vec{m}\left(3\right)+\vec{m}\left(6\right)+\vec{m}\left(9\right)+\vec{m}\left(18\right)\right]\cdot \frac{1}{4}$$

(4) $$S_{7}=\left[\vec{m}\left(7\right)+\vec{m}\left(14\right)\right]\cdot \frac{1}{2}$$

A salience value for each conceivable meter is constructed, i.e., double, triple, and septuple, as shown in Equations (3)–(5), respectively. The ultimate meter of the song is determined by the sum of S2, S3, and S7.

Holzapfel and Stylianou in [75] set out to estimate the rhythmic similarities in Turkish traditional music and on this path, the time signature was estimated with a data set consisting of 288 songs distributed along the six classes of different rhythmic schemes (9/8, 10/8, 8/8, 3/4, 4/4, 5/8). Although this was not the aim of this research, he proposed a method for estimating the time signature because the overall study was compared to a start-of-the-art estimation technique which Like Uhle proposed in [42]. The onset periods are read from the MIDI files, and each onset is allocated a weight. After evaluating several strategies for assigning weights, the most popular scheme was adopted: the weight of an onset may be compared to the note length, to melody characteristics, or all onsets are assigned the same weight. To evaluate a piece’s time signature, all pairwise dissimilarities between songs were computed using either the scale-free auto correlation function (ACF) or the STM vectors, and a cosine distance; a similar method was used in [87]. The same method used by Brown in [88] since it is a count of the number of events that occur during an occurrence at time zero if events are clustered from measure to measure, with a higher occurrence of an event happening with the measure’s time isolation, therefore peaks in the auto-correlation function should show the periods when measurements begin [89]. A single melody line was extracted from the music score for analysis. This produced dissimilarity matrices with values close to zero when two parts were discovered to be alike in terms of rhythmic information. The accuracy of an updated k-Nearest Neighbor (kNN) classification was calculated in order to calculate the consistency of the proposed rhythmic similarity metric [90,91,92,93]. The power of a similarity matrix in this sphere lies with the distance between the notes in comparison. That is, the higher the distance, the lesser the similarity and vice versa. Hence the need to evaluate the impact of the value of K on the nearest neighbor. Each individual song was then used as a query for classification into one of the available groups. The dissimilarity matrix was classified using the modified kNN. The melodic line *x*[*n*] was subjected to a short time auto-correlation calculation defined as

(5) $$A\left[m\right]=\sum_{n=0}^{N-1}x\left[n\right]x\left[n+m\right]$$

where the average is taken over *N* samples and m is the auto-correlation time in samples.

Coyle and Gainza in [74] proposed a method to detect the time signature from any given musical piece by using an Audio Similarity Matrix (ASM). The ASM compared longer audio segments (bars) from the combination of shorter segments (fraction of a note). This was based on an assumption that musical pieces have repetitive bars at different parts. A spectrogram with a frame length equal to a fraction of the duration of the song’s beat was generated using prior knowledge of the song’s tempo; a technique asserted by Kris West in [94]. Following that, the song’s first note was obtained. The reference ASM was then produced by taking the Euclidian distance between the frames beginning with the first note and this enables the parallels between minor musical incidents such as short notes to be captured. Then, a multi-resolution ASM technique is used to create other audio similarity matrices representing different bar lengths. After computing all of the ASMs within a certain range, the ASM with the greatest resemblance between its components would conform to the bar duration and a technique for detecting the song’s anacrusis—an anticipatory note or notes occurring before the first bar of a piece, is added. Finally, the time signature is estimated, as well as a more precise tempo measurement.

The music meter estimation problem can also be considered as a classification task as demonstrated by Varewyck et al. in [78]. Having considered the previous methods in this field that worked, they used the Support Vector Machine (SVM) for this purpose. Prior to the periodicity analysis, an external beat tracker was used to perform beat-level analysis, alongside, spectral envelope and pitch analysis were also carried out. Furthermore, a similarity analysis of the interval between two successive beats which they called Inter-Beat-Interval (IBI) already shown by Gouyon and Herrera (2003) [72] was performed. Hereafter, a hypothesis for the meter generated was developed and the meter was obtained. The similarity of the IBI was calculated using cosine similarity as shown in the equation below

(6) $$CS\left(b\right)=\frac{\langle \vec{z}\left(b-1\right),\vec{z}\left(b\right)\rangle }{∥\vec{z}\left(b-1\right)∥∥\vec{z}\left(b\right)∥}$$

where *b* is the beat, $\vec{z}\left(b-1\right)$ and $\vec{z}\left(b\right)$ are low dimensional vectors grouped by related features. Eventually, they created an automatic meter classification method with the best combination of features that made an error of around 10% in duple/triple meter classification and around 28% in meter 3, 4, and 6 with a balanced set of 30 song samples.

Meter estimation for traditional folk songs is especially more challenging as much research is usually carried out on Western music. However, Estefan et al. in [79] made some attempt to estimate the meter and beat of Colombian dance music known as the bambuco. The bambuco has a superposition of $\frac{3}{4}$ and $\frac{6}{8}$ m but due to the caudal syncopation and the accentuation of the third beat, the case of downbeat does not hold for this type of music. With the ACMUS-MIR dataset (V1.1), a collection of annotated music from the Andes region in Colombia, they were able to perform beat tracking and meter estimation. For the study, 10 candidates were asked to tap to the rhythm in order to choose 10 bambuco packs with Sonic Visualiser’s on the computer keyboard. There were two sets of annotations: (1) beats were taped while the audio was playing (without any visual information) and participants were not granted permission to make any adjustments. (2) Participants were permitted to change the Sonic Visualiser’s first beat annotations using both audio and audio waveform visuals. Three musicologists from Colombia evaluated the beat annotations from these 10 participants in order to establish the underlying meters of each track. Each annotation was mapped directly to a certain meter, either $\frac{3}{4}$, $\frac{6}{8}$, or a combination; even though the participants were asked to naturally tap to the beats. They also performed beat tracking using two methods; madmon and multiBT while evaluating the F1 score for each perceived meter. For $\frac{3}{4}$, madmon had 76.05% while multiBT had 42.79% and for $\frac{6}{8}$, madmon had 41.13% while multiBT had 45.15%. In conclusion, in the annotations made by the research participants, five metric alternatives were discovered.

## 5. Deep Learning Techniques

Things are a little different with deep learning, because more knowledge is gathered. With deep learning, it is basically a neural network with three or more layers. Although a single-layer neural network may still generate approximate predictions, more hidden layers can assist optimize and tune for accuracy. In resolving several complicated learning issues, such as sentiment analysis, extraction of functions, genre classification, and prediction, Convolutional Neural Networks (CNNs) have been used extensively [95,96,97]. For tempo data such as audio signals and words sequencing, a hybrid model of CNNs and Recurrent Neural Networks (RNNs) was recently used [98]. Audio data is represented by frameworks and the sequential character of audio is entirely overlooked in the traditional RNN approach for temporal classification, hence the need for a well-modeled sequential network; the long-term recurrent neural network (LSTM) which has recorded successes for a number of sequence labeling and sequence prediction tasks [99,100]. Convolutional-Recurrent Neural Networks (CRNNs) are complicated neural networks constructed by the combination of CNN and RNN. As an adapted CNN model, the RNN architecture is placed on CNN structure with the aim of obtaining local features using CNN layers and temporal summation by RNN networks. The main components for a CNN network are: input type, rate of learning, batches and architectural activation features, and the ideal type of input for music information collection is the mel-spectrogram [97]. Mel spectrograms are comprised of broad functionality for latent feature learning and onset and offset detection since the Mel scale has been shown to be similar to the human auditory system [81,101]. In order to obtain a mel-spectrogram signal, the pre-processing phase is necessary for STFT (Fourier short transform) and the log amplitude spectrogram. The methods in this section discussed and summarized in Table 4 consist of neural networks that extract the time signature as a feature that can be used as input for further calculation or classification problems in the MIR domain rather than estimating it exactly. Handcrafted features like Mel Frequency Cepstral Coefficients (MFCC), Statistical Spectrum Descriptors (SSD), and Robust Local Binary Patterns (RLBP) [102], used in deep learning are extracted based on human’s domain knowledge [101]. However, these features have not been totally proven to be correlated to meter or time signature detection and their effectiveness and validity are not very clear.

Rajan et al. in [108] proposed a meter classification scheme using musical texture features (MTF) with a deep neural network and a hybrid Gaussian mixture model-deep neural network (GMM-DNN) framework. The proposed system’s performance was assessed using a freshly produced poetry corpus in Malayalam, one of India’s most widely spoken languages, and compared to the performance of a support vector machine (SVM) classifier. A total of 13 dim MFCCs were extracted using frame-size of 40 ms and frame-shift of 10 ms alongside seven other features; spectral centroid, spectral roll-off, spectral flux, zero crossing, low energy, RMS, and spectrum energy. Rectified linear units (ReLUs) were chosen as the activation function for hidden layers, while the softmax function was used for the output layer. These methods produce an accuracy of 86.66 percent in the hybrid GMM-DNN framework. The overall accuracies for DNN and GMM-DNN were 85.83 percent and 86.66 percent, respectively.

### 5.1. Convolutional Neural Netorks

**Figure 4:**
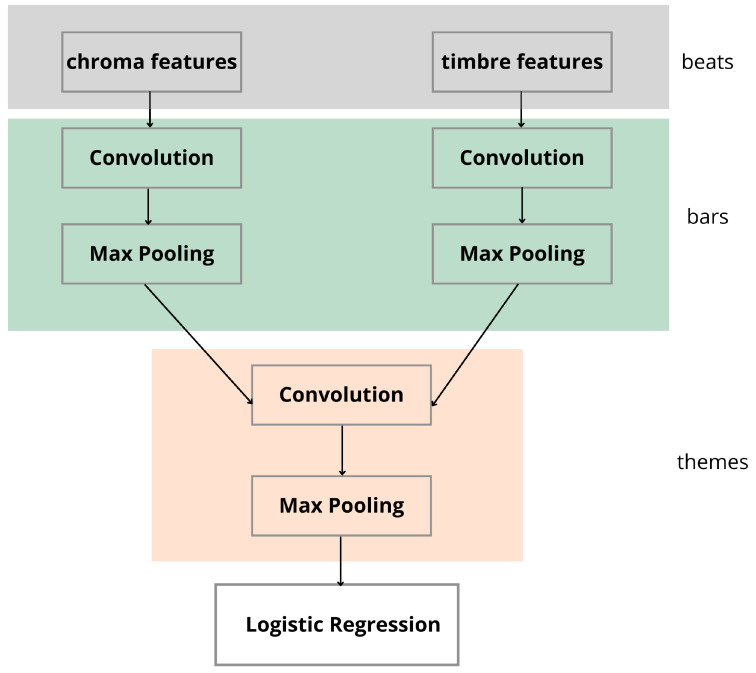
The convolutional neural network architecture block diagram with two kinds of input features (chroma and timbre).

In a study conducted by Sander Dieleman et al. in [103] where unsupervised pre-training was performed using the Million Song Dataset, the learnt parameters were transferred to a convolutional network with 24 input features. Timbre properties from the dataset were presented to the network as shown in Figure 4. Two input layers composed of chroma and timbre characteristics were stacked with separate convolution layers and the output of these layers was then maxpooled. The performance of the max-pooling layer was invariant to all displacements of less than one bar (up to 3 beats). The accuracy in terms of time signature was not stated since this was not the main objective of the research.

Sebastian Böck et al. [105] showed that tempo estimation can be achieved by learning from a beat tracking process in a multi-task learning algorithm since they are highly interconnected; a method that has been used in other research areas for optimization [109,110]. This approach proved effective in that mutual information of both tasks was brought forward by one improving the other. The multi-task approach extends a beat tracking system built around temporal convolutional networks (TCNs) and feeds the result into a tempo classification layer. Instead of using raw audio as data input, dilated convolutions are applied to a heavily sub-sampled low-dimensional attribute representation. This 16-dimensional function vector is generated by adding several convolution and max pooling operations to the input audio signal’s log magnitude spectrogram. The log magnitude spectrum is obtained because this is what the human ear can perceive [111,112]. The spectrogram is produced using a window and FFT size of 2048 samples, as well as a hop size of 441 samples. The convolutional layers each have 16 filters, with kernel sizes of 3×3 for the first two layers and 1×8 for the final layer. The method was tested on a variety of existing beat- and tempo-annotated datasets, and its success was compared to reference systems in both tasks. Findings show that the multi-task formulation produces cutting-edge efficiency in both tempo estimation and beat recording. The most noticeable improvement in output occurs on a dataset where the network was trained on tempo labels but where the beat annotations are mostly ignored by the network. The underlying beat tracking system is inspired by two well-known deep learning methods: the WaveNet model [38] and the latest state-of-the-art in musical audio beat tracking, which employs a bi-directional long short-term memory (BLSTM) recurrent architecture. To train the system, annotated beat training data as impulse were represented at the same temporal resolution as of the input feature (i.e., 100 frames per second) and different datasets were used for this training and eventual evaluation, unlike other approaches where one single dataset is divided into training and test sets.

Tracking meter at a higher metrical level is a task pursued under the title of downbeat detection [113]. Therefore we can also consider downbeat detection with deep learning features. Durand and Essid in [104] suggested a random field method conditioning an audio signal’s downbeat. In the first instance the signal generated four additional characteristics pertaining to harmony, rhythm, melody, and bass, and the tatum level was separated. Adapted convolutional neural networks (CNN) were then used for feature learning based on each feature’s characteristics. Finally, a feature representation concatenated from the networks’ final and/or penultimate layers was used to describe observation feature functions and fed into a Markovian model of Conditional Random Field (CRF) that produced the downbeat series. The model was evaluated using a Friedman’s test and a Tukey’s honestly significant criterion (HSD) and was found to have a F-measure improvement of +0.9% using features from the last layer and 95% confidence interval.

**Figure 5:**
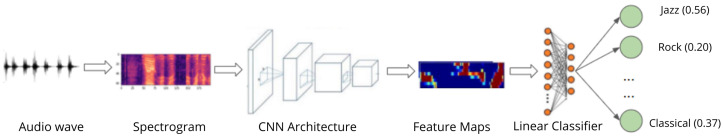
A typical convolutional neural network architecture used to time signature detection—audio signal processed into spectrogram which is an input to convolutional layers, and then an outcome is an input to classical artificial neural network.

With a deep learning approach, music domain assumptions are relevant when not enough training data are available as suggested by Pons et al. in a study done recently in 2017 [69]. They were able to automatically categorize audio samples using waveforms as input and a very small convolutional filter on a convolutional neural network—a common architecture for music genre classification as shown in Figure 5 and thus indirectly calculated various attributes, one of which was the meter. The CNN architecture was divided into input, front-end, back-end, and output for easy implementation. The front-end that takes in the spectrogram is a single-layer CNN with multiple filter shapes divided into two branches: top branch—timbral features, and lower branch—temporal features. The shared backend is made up of three convolutional layers (each with 512 filters and two residual connections), two pooling layers, and a dense layer. With two models combined where one implemented classical audio features extraction with minimal assumption and the other dealt with spectrograms, and a design that heavily relies on musical domain knowledge, meter tags were obtained.

This kind of pipeline was also suggested by Humphrey et al. [114] where it was advocated to move beyond feature design to automatic feature learning. A fraction of the Million Song Dataset alongside the MagnaTagATune (25 k songs) which have been mentioned in the dataset section, and a private dataset of 1.2 M songs were combined together to validate the two distinct music auto-tagging design approaches considered. The result of this study brought about an approach to learn timbral and temporal features with 88% accuracy.

Purwins et al. in [106] showed how deep learning techniques could be very useful in audio signal processing in the area of beat tracking, meter identification, downbeat tracking, key detection, melody extraction, chord estimation, and tempo estimation by processing speech, music, and environmental sounds. Whereas in traditional signal processing, MFCCs are the dominant features; in deep learning the log-mel spectrograms (see Section 1) are the pre-dominant features. As confirmed by Purwmins, the convolutional neural networks have a fixed flexible field, which limits the temporal context taken into account for a prediction while also making it very simple to expand or narrow the context used. While it was not explicitly stated which of the three popular methods of deep learning performs the best, the data used sometimes determines the method to be used. For this analysis, the Million Song Dataset was chosen to reduce a 29 s log-mel spectrogram to an 1 × 1 feature map and categorized using 3 × 3 convolutions interposed with max-pooling which yielded a good result of 0.894 AUC.

### 5.2. Convo-Recurrent Neural Networks

Fuentes et al. in [107] combined a non-machine learning approach as well as deep learning to estimate downbeat and in the process extract the time signature. The deep learning approach was a combination of a convolutional and recurrent network which they called CRNN proposed in their previous work [115]. By using the Beatles dataset because of its peculiarity in annotated features such as beats and downbeats, they considered a set of labels Y which represents the beat position inside a bar, then took bar lengths of 3 and 4 beats, corresponding to 3/4 and 4/4 m. The output labels *y* are a function of two variables: the beat position $b\in B=\{1,\ldots ,b_{max}\left(r\right)\}$ and the number of beats per bar $r\in R=\{r_{1},\ldots ,r_{n}\}$, which relates to the time signature of the piece. The model experienced a level of success but it was incapable of identifying rare music variations in order to fit the global time signature consistently. For example, it estimated more 4/4 pieces than 3/4. Consequently, this model improves the downbeat tracking performance of the mean F-measure from 0.35 to 0.72.

## 6. Conclusions and Future Pathways

**Figure 6:**
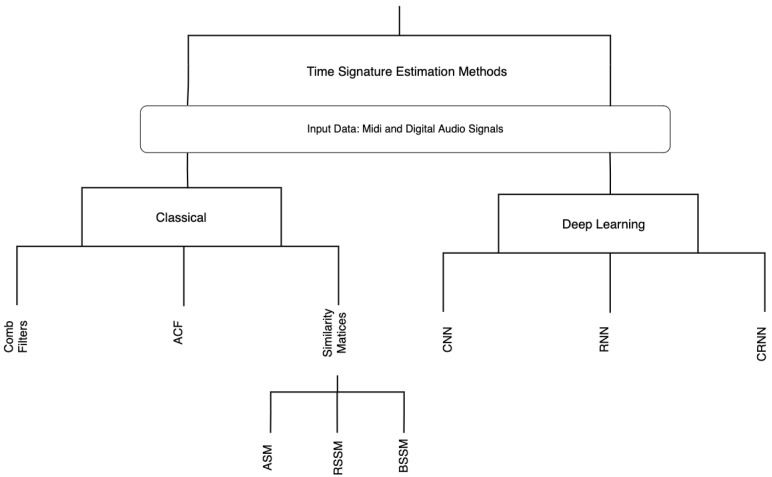
A dendrogram of both classical and deep learning techniques discussed in this paper.

In this paper, we presented a summary of different methods for estimating time signature in music, considering both state-of-the-art classical and deep learning methods with a focus on the dataset used and the accuracy obtained as shown on the dendrogram in Figure 6. Since there has not been a study like this, there is a need for this analysis. The history of datasets has also been explored in terms of their production processes and purposes. The experiments that have been conducted so far have produced promising findings, indicating that time signature may be a significant feature of music genre classification.

This survey has shown that in order to estimate the time signature using digital signal processing analysis, the most promising approach has come from generating some similarity matrices of the temporal features of audio or MIDI files when music signal is converted into an appropriate feature sequence. Based on a similarity measure, a self-similarity matrix is generated from the feature sequence. The SSM generates blocks and pathways with a high overall score. Each block or path specifies a pair of segments that are comparable. Using a clustering step, whole groups of mutually comparable segments are generated from the pairwise relations. A more detailed research into similarity matrices of MFCCs between 4 and 20 could yield better results. It is important to note that the ASM, RSSM, BSSM, and ACF work better on MIDI files than on digital audio files, however, MIDI files are not popularly used anymore. With audio samples, time signature estimation becomes relative to the tempo of the track which these other methods did not take seriously. In terms of using any deep learning approach, network architectures such as RNN has shown some level of success but cannot retain audio information for too long, however, the CNN architecture is definitely the way forward in this kind of task because it gives more accuracy for a wide range of both regular and irregular time signatures but it also takes more computational time and power to perform this task. A combination of two architectures like CNN and RNN where features are extracted in the convoluted layer and later transferred to recurrent layer has also proven to be effective in time-based series of audio signals. This implies that transfer learning—an approach that has not been fully explored in this research area could also be given more attention.

More than 70% of the studies considered in this review assumed that music pieces had repeated bars at various points in the piece, which is not always the case. Estimating musical parts with an irregular signature or beat is challenging. As a result, additional research may be conducted in this field. The aim of this analysis is to chart a course for future study in feature extraction of machine learning algorithms used in music genre classification, time signature estimation and identification, and beat and tempo estimation in the Music Information Retrieval domain. Using a better approach as a pre-processor to retrieve the time signature as an additional feature in a neural network pipeline could drastically increase the accuracy of the model eventually.

## Figures and Tables

**Table 1:** Differences between the input signals.

Criteria	MIDI	Digital Audio
Definition	A MIDI file is a computer software that provides music info.	A digital audio refers to digital sound reproduction and transmission.
Pros	Files of small size fit on a disk easily. The files are perfect at all times.	The exact sound files are reproduced. It replicates superior quality.
Cons	There is variation from the original sound.	They take more disk space with more minutes of sound, files can get corrupted with a little manipulation.
Format Type	Compressed.	Compressed.
Information Data	Does not contain any audio information.	Contains recorded audio information.

**Table 2:** Datasets and their statistics.

Dataset Name	Year Created	Number of Samples	Data Samples
RWC [49]	2002	365	Audio
CAL500 [50]	2008	502	MIDI
GZTAN [51]	2002	1000	Audio
USPOP [52]	2002	8752	MIDI
Swat10K [53]	2010	10,870	MIDI
MagnaTagATune [54]	2009	25,863	Audio
FMA [55]	2016	106,574	Audio
MusicCLEF [56]	2012	200,000	Audio
MSD [57]	2011	1,000,000	CSV

**Table 3:** Summary of classical estimation methods.

Year	Method	Dataset	Data	Accuracy (%)
2003	SVM [72]	Self generated	Audio	83
2003	ACF [42]	Excerpts of percussive music	Audio	73.5
2004	SSM [73]	Greek music samples	Audio	95.5
2007	ASM [74]	Commercial CD recordings	Audio	75
2009	ACF, OSS [75]	Usul	MIDI	77.8
2009	BSSM, ASM [76]	Generated samples	Audio	95
2011	Comb Filter [77]	Indian Music DB	Audio	88.7
2013	SVM [78]	Generated Samples	Audio	90
2014	RSSM [47]	MIDI keyboard scores	MIDI	93
2020	Annotation Workflow [79]	ACMUS-MIR	Audio	75.06

**Table 4:** Summary of deep learning estimation methods.

Year	Method	Dataset	Accuracy (%)
2011	CNN [103]	MSD	Not stated
2016	CNN [104]	Multiple datasets	90
2017	CNN [69]	MSD, MagTagATune	88
2019	TCN [105]	Annotated dataset	93
2019	CNN [106]	MSD	89
2019	CRNN [107]	Beatles	72
2019	GMM-DNN [108]	Poetry corpus	86

## Data Availability

Not applicable.
